# Emerging strategies for targeting vasculogenic mimicry in breast cancer treatment

**DOI:** 10.1007/s12672-025-04266-5

**Published:** 2025-12-16

**Authors:** Nare Sekoba, Demetra Demetriou, Nkhensani Chauke-Malinga, Peace Mabeta

**Affiliations:** 1 Department of Natural and Rehabilitative Sciences, Faculty of Health Sciences, University of Fort Hare, East London, South Africa; 2 Angiogenesis Laboratory, Department of Physiology, Faculty of Health Sciences, University of Pretoria, Pretoria, South Africa; 3 SAMRC Precision Oncology Research Unit (PORU), DSI/NRF SARChI Chair in Precision Oncology and Cancer Prevention (POCP), Pan African Cancer Research Institute (PACRI), University of Pretoria, Pretoria, South Africa; 4 Papillon Aesthetics, Linksfield, Netcare, Johannesburg, South Africa

**Keywords:** Breast cancer, Tumor vascularization, Therapeutic targeting, Vasculogenic mimicry, Angiogenesis

## Abstract

Breast cancer represents the most commonly diagnosed malignancy worldwide and is the fourth leading cause of cancer-related mortality. Approximately 2.3 million new cases of breast cancer were diagnosed worldwide in 2022, accounting for 11.6% of all cancer cases, with approximately 670,000 associated deaths. Breast tumors frequently present with abnormal and highly vascularized networks, promoting accelerated growth and contributing to metastatic potential. Increased vascularization often indicates a more aggressive cancer and significantly affects breast cancer treatment. While angiogenesis offers potential therapeutic targets, it also complicates treatment strategies. Anti-angiogenic drugs such as bevacizumab, which target vascular endothelial growth factor-A signaling, have shown potential in limiting tumor growth. However, their success has been limited, as tumors can develop resistance through alternative pathways. Aggressive breast cancer cells, regardless of estrogen-receptor status, can form vessel-like structures through vasculogenic mimicry. This phenomenon also enables them to evade anti-angiogenic treatment and contributes significantly to tumor resistance to various therapeutic interventions. Moreover, vasculogenic mimicry-positive breast cancer patients exhibit high tumor grade, increased invasiveness, metastasis, and poorer survival outcomes as compared to vasculogenic mimicry-negative breast cancer patients. At present, there are no clinically approved therapies that specifically target vasculogenic mimicry in breast cancer. The intricate molecular mechanisms involved in vasculogenic mimicry within breast cancer present considerable challenges to the development of effective therapeutic strategies. Achieving therapeutic breakthroughs that address this phenomenon would represent a major step in the management of breast cancer. This review examines key molecular pathways that regulate vasculogenic mimicry in breast cancer and assesses the potential of targeting vasculogenic mimicry for therapeutic intervention.

## Introduction

Breast cancer is the leading cause of cancer-related mortality among women worldwide [1]. According to the International Agency for Research on Cancer (IARC) and World Health Organization (WHO), on average 1 in 20 women worldwide will be diagnosed with breast cancer during their lifetime [2]. Several risk factors have been associated with breast cancer, including obesity, extended exposure to estrogen, reproductive history, and genetic predispositions such as mutations in the BRCA genes [3, 4]. Notably, about 50% of women diagnosed with breast cancer do not have identifiable risk factors, apart from being female and over the age of 40, supporting regular screening and early detection [4, 5].

Pathologically, breast cancer arises predominantly from the epithelial lining of the ducts (≈ 85%) and less commonly from the lobules (≈ 15%). Clinical assessment relies on both morphological features (nuclear grade, tubular grade, mitotic index, and architectural characteristics) and clinicopathological parameters (tumor size, lymph node involvement, and metastasis), which together guide prognosis and therapeutic decision-making [6, 7].

Molecular classification based on gene expression profiling has further refined breast cancer diagnosis. Tumors are categorized by the presence or absence of estrogen receptor (ER), progesterone receptor (PR), and human epidermal receptor 2 (HER2) [8, 9]. Luminal A tumors (ER+/PR+/HER2−) and Luminal B tumors (ER+/PR+/HER2+) are hormone-receptor positive, while HER2-enriched tumors (ER−/PR−/HER2+) and basal-like or triple-negative breast cancers (TNBC; ER−/PR−/HER2−) represent more aggressive subtypes. Breast cancer is also stratified clinically into premenopausal (< 50 years) and postmenopausal (>50 years) groups, each with distinct biological profiles and outcomes [10].

Depending on the stage, subtype, and pre- or post-menopausal status of the disease, surgery is the standard clinical treatment for breast cancer. This is followed by radiation and systemic medication therapy. The current management of breast cancer patients is determined by luminal status, HER2 enrichment, or triple-negative status. Breast tumors that are ER and PR positive are treated with target hormone therapy, such as tamoxifen and an aromatase inhibitor, while HER2-positive breast cancer is treated with a monoclonal antibody drug, trastuzumab [11]. Chemotherapy has been the primary option for treating TNBC. Although hormonal, chemotherapy, and targeted therapy have been used over the years to manage breast cancer, metastasis, recurrence, and relapse due to treatment resistance have been evident in a vast number of patients [12]. This resistance underscores the role of alternative tumor survival mechanisms, such as the formation of new blood vessels through angiogenesis, which supplies oxygen and nutrients to sustain tumor growth [13]. However, even anti-angiogenic mono-therapies have led to resistance and relapse, highlighting the complexity of tumor progression mechanisms that extend beyond conventional angiogenesis [14, 15].

Emerging evidence suggests that breast cancer cells can exploit additional adaptive strategies to secure nutrient supply and facilitate metastasis. One such mechanism is vasculogenic mimicry (VM), in which highly plastic tumor cells form vessel-like networks independent of endothelial cells [16, 17]. Initially described in aggressive melanoma, VM has since been observed in several malignancies, including breast cancer, where it correlates with poor prognosis, metastasis, and resistance to anti-angiogenic therapies [16, 18, 19].

Understanding the regulatory mechanisms and clinical implications of VM in breast cancer is essential for improving therapeutic strategies. This review, therefore, explores VM across molecular subtypes of breast cancer, emphasizing its regulators, detection methods, and emerging therapeutic interventions. Variability in VM definitions, detection methods, and experimental models across studies has limited direct comparison of findings. Moreover, most available evidence is derived from preclinical systems, which may not fully represent human breast tumors. By emphasizing recent developments and acknowledging persistent challenges, we seek to establish VM as an important yet often overlooked element in breast cancer progression, while examining emerging strategies for targeting this process to enhance patient outcomes.

## Vasculogenic mimicry in different subtypes of breast cancer

Vasculogenic mimicry exhibits variable expression across breast cancer molecular subtypes, reflecting the disease’s inherent heterogeneity. It is most prevalent in aggressive subtypes such as TNBC, but rare in less aggressive luminal types [20, 21]. This pattern suggests that VM formation is driven by molecular processes that promote aggressive tumor behavior and invasiveness [20, 22].

### Triple-negative breast cancer

**Fig. 1:**
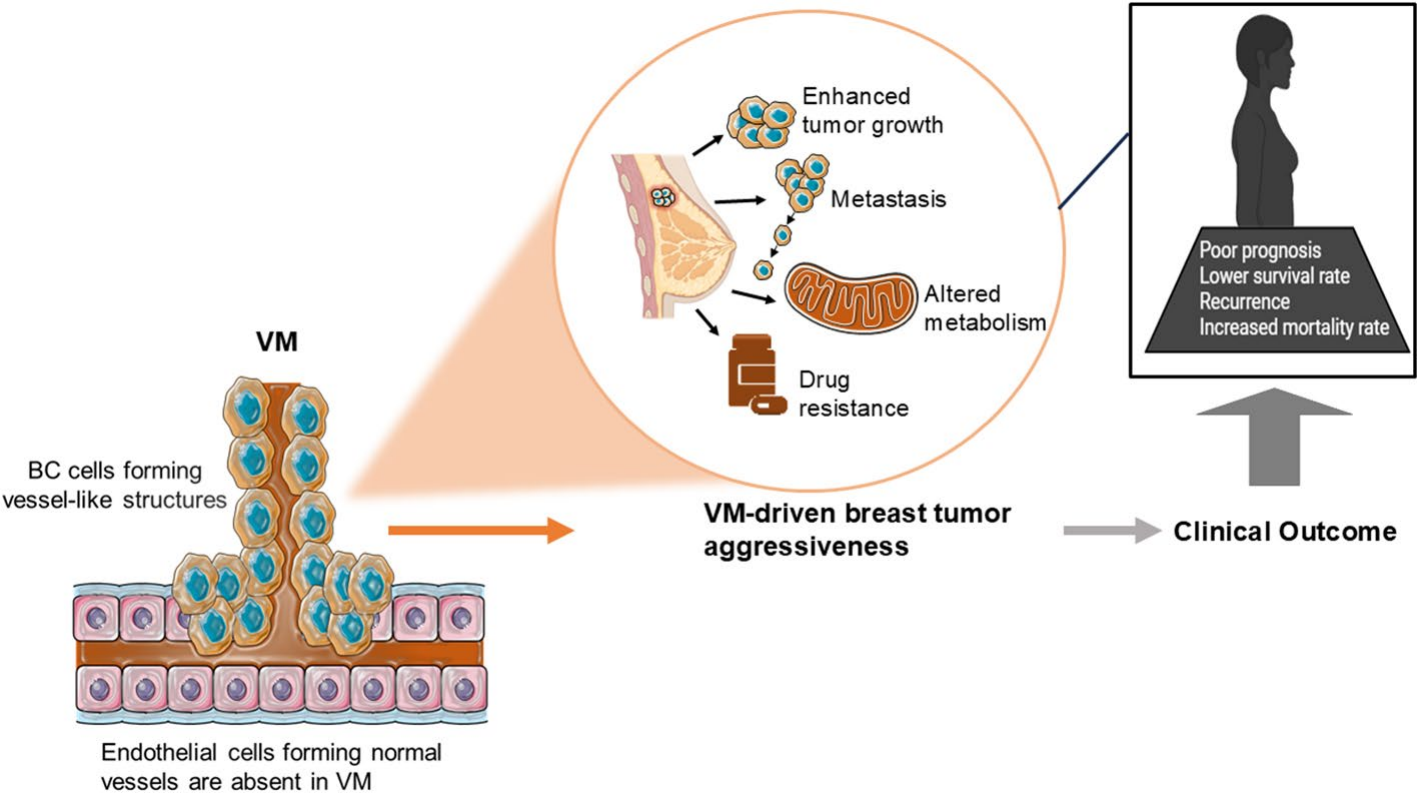
The formation of microcirculatory vessels by aggressive breast tumors. Breast cancer (BC) cells can form vessel-like channels through vasculogenic mimicry (VM), a process independent of endothelial cells and distinct from classical angiogenesis. These VM structures facilitate the supply of nutrients and oxygen, promoting tumor growth, metastasis, altered metabolism, and drug resistance. Clinically, VM is associated with poor prognosis, lower survival rates, increased recurrence, and higher mortality in breast cancer patients. Drawn using drawing tools. Abbreviations: BC, Breast Cancer; VM, Vasculogenic mimicry

Triple-negative breast cancer exhibits high proliferative activity, and numerous preclinical studies have associated TNBC cells with the ability to form VM structures, which may facilitate tumor cell dissemination and invasion **(**Fig. 1**)** [20, 22, 23]. Consequently, TNBC cell lines, such as MDA-MB-231 and Hs578T, are frequently used to investigate the molecular mechanisms and potential therapeutic strategies targeting VM [24–26]. While these studies have provided valuable insights, it is essential to acknowledge that VM is heterogeneous across breast cancer subtypes and even within tumors; therefore, it should be approached with consideration of this variability.

### HER2-positive breast cancer

While HER2-positive breast cancers also exhibit aggressive phenotypes, few studies have investigated VM formation in this subtype [27]. In an *in vitro study*, trastuzumab-resistant HER2-positive SKBR3 cells could form endothelial-independent tubular structures, in contrast to the parental HER2-positive SKBR3 breast cancer cells [28]. Treatment with trastuzumab induces repolarization of M2 to M1 macrophages, promoting an anti-tumor immune response through the release of pro-inflammatory cytokines such as tumor necrosis factor (TNF)-α and interleukin (IL)-6 [29, 30]. However, sustained TNF-α signaling in the tumor microenvironment has been shown to induce MUC4 expression, which masks the HER2 epitope and reduces trastuzumab efficacy, thereby facilitating immune evasion [31, 32]. Bruni et al. demonstrated that blocking soluble TNF-α downregulates MUC4 and shifts tumor-associated macrophages toward an M1 phenotype, highlighting the role of TNF-α in maintaining an immunosuppressive microenvironment in resistant tumors [31, 33]. We hypothesize that TNF-α-mediated immunosuppression may indirectly create a microenvironment permissive to VM. Collectively, these observations suggest that VM in HER2-positive cancers may represent an adaptive response to therapeutic resistance rather than a baseline feature of the subtype.

### Luminal breast cancer

Luminal breast cancer cells, typically less invasive, rarely form VM structures under normal conditions [21, 34, 35]. However, under specific stimuli such as HER2 overexpression or drug resistance, luminal cells can acquire VM capability. For example, transfection with the pcDNA3-HER2 mammalian expression plasmid induces vascular endothelial (VE)-cadherin expression (a regulator of VM) and vessel-like structures, while Adriamycin-resistant luminal breast cancer cells display similar features [34, 36]. Exposure to interleukin-1β or hypoxic conditions also promotes VM by upregulating vascular endothelial growth factor receptor (VEGFR)-1, matrix metalloproteinase (MMP)9, and MMP2 [21, 37, 38]. These findings suggest that luminal cells may possess latent plasticity that can be unmasked by therapeutic stress or microenvironmental changes, indicating that VM can represent a reversible, adaptive phenotype under specific conditions rather than a fixed trait.

### Clinical evidence

Preclinical studies employing patient specimens further support the presence of VM across all molecular subtypes, with the highest prevalence in TNBC (≈ 49–67%), intermediate prevalence in HER2-positive tumors (≈ 13–24%), and lowest prevalence in luminal tumors (≈ 19%) [39, 40]. Variety in VM findings among studies often stems from methodological heterogeneity and a lack of adjustment for clinicopathological variables.

For example, Liu et al. analyzed 120 invasive breast cancer specimens collected between 2004 and 2007, using CD31/periodic acid-Schiff (PAS) double-staining, and reported VM in 22.5% of HER2-positive cases [36]. In a comparable sample size of invasive ductal carcinoma collected between 2011 and 2016, Jafarian et al. identified VM in 13.3% of HER2-positive patients through immunohistochemistry [41]. The absence of histochemical validation in the latter study may contribute to its lower VM detection rate, as CD31/PAS double-staining appears more sensitive in detecting VM compared to immunohistochemistry alone. Neither Liu nor Jafarian et al. adjusted for clinicopathological parameters, such as tumor grade, lymph node status, and size, despite analyzing cohorts of invasive breast cancer specimens that varied in clinical characteristics. In a subsequent study, Liu et al. analyzed 90 invasive ductal carcinoma cases (collected between 1998 and 2005) and incorporated clinicopathological parameters into multivariate survival models [42]. Although HER2 status emerged as a significant predictor of disease-free survival, it was not significantly associated with VM formation, supporting the view that VM in HER2-positive cancers may represent an acquired, stress-responsive phenomenon.

Overall, VM prevalence variation reflects a combination of biological and technical methodology factors rather than intrinsic subtype identity.

### Tumor microenvironment and intratumoral heterogeneity

There is significant heterogeneity within breast cancer subtypes, driven by complex interactions between tumor cells and other components of the tumor microenvironment [43]. Insights from heterocellular gene signatures, originally developed from colorectal cancer, representing different cell types (stem-like, stromal, mesenchymal, immune, and epithelial), have prompted researchers to explore similar cellular diversity within breast cancer subtypes.

This analysis revealed that luminal A, previously considered a relatively homogeneous and low-risk subtype, can be further divided into stem-like and inflammatory phenotypes characterized by high expression of immune checkpoint and immune-related genes. These findings suggest that even within estrogen receptor-positive cancers, there exist immune-enriched subtypes that may benefit from immunotherapy. Similarly, HER2-positive and basal-like subtypes (which include most TNBCs) often show enrichment in immune gene signatures consistent with an inflammatory heterocellular profile [44]. This refined stratification highlights important differences in tumor biology and microenvironment that are not captured by conventional classifications. Such distinctions could guide more personalized treatment approaches, particularly the potential use of immunotherapy in immune-enriched subtypes and improve prognostic accuracy by identifying high-risk patients within generally low-risk categories.

This growing appreciation of intratumoral heterogeneity also has implications for the detection of VM. Patient-derived tissue samples reveal inter-patient variability that may explain why VM is observed in some TNBC cases but not others. Furthermore, heterogeneity within the TME, as well as interactions among its components, may contribute to differences in observations of VM between *in vitro *and *ex vivo *models of HER2-positive and luminal breast cancer. *In vitro *models that are typically based on monocultures do not recapitulate the cellular diversity or microenvironmental complexities present in patient-derived tissues [45]. Thus, accurate modeling of the tumor microenvironment is crucial for elucidating VM regulation across breast cancer subtypes.

### Angiogenesis and vasculogenic mimicry in breast cancer

Tumor angiogenesis and VM represent distinct but complementary mechanisms of vascularization in breast cancer. Structurally, angiogenesis results in blood vessels lined by endothelial cells, supported by pericytes, and a basement membrane. In contrast, VM channels are lined by tumor cells and lack true endothelial or perivascular support [46, 47]. Functionally, angiogenic vessels integrate into the host circulation to sustain nutrient delivery, while VM establishes tumor cell–derived, perfusable networks that maintain oxygenation under hypoxic or anti-angiogenic stress [48].

Molecularly, angiogenesis is driven by classical angiogenic factors (e.g., vascular angiogenic growth factor-A (VEGF-A), fibroblast growth factor), while VM involves tumor cell plasticity, expression of endothelial markers (e.g., VE-cadherin), MMP activity, and epithelial-to-mesenchymal (EMT)-related pathways [20, 46, 48]. These differences highlight the necessity for distinct detection methods and therapeutic approaches.

Accurate identification of both angiogenesis and VM in breast cancer specimens is critical for risk stratification and treatment planning. Histological and immunohistochemical techniques are employed to distinguish between the two processes [16].

The presence of VM in breast cancer is associated with higher tumor grade, increased metastatic potential, and reduced survival, making it a valuable prognostic marker. Therapeutically, a dual approach targeting both angiogenesis and VM may be necessary for effective disease control in aggressive breast cancer subtypes. Ongoing research into the molecular underpinnings of VM holds promise for developing novel, more effective therapies.

## Molecular and microenvironment regulation of vasculogenic mimicry in breast cancer

The formation of VM in breast cancer is governed by a complex interplay of molecular and microenvironmental regulators that confer endothelial-like properties to tumor cells. These mechanisms promote tumor plasticity, invasion, and metabolic adaptability. The main regulatory axes include hypoxia-driven signaling, VM execution pathways, and metabolic reprogramming.

### Hypoxia as the central driver of vasculogenic mimicry

Hypoxia, a defining feature of the tumor microenvironment, upregulates hypoxia-inducible factor (HIF)-1α, which translocates into the nucleus and dimerizes with the HIF-1β subunit to activate downstream genes associated with VM formation [49]. Hypoxia-inducible factor-1α activation promotes breast cancer stem cell (CSC) phenotype, a key hallmark of VM. An *in vitro* study demonstrated that hypoxia enhances the CD133⁺ CSC subpopulation in MDA-MB-231 cells through a TWIST 1-dependent mechanism, enabling these cells to form VM channels [22]. Similarly, breast CSCs are induced through the epidermal growth factor receptor signaling pathway to form VM channels [50, 51]. The capacity of a tumor to acquire a CSC phenotype facilitates its survival and adaptation within a hypoxic environment.

**Fig. 2:**
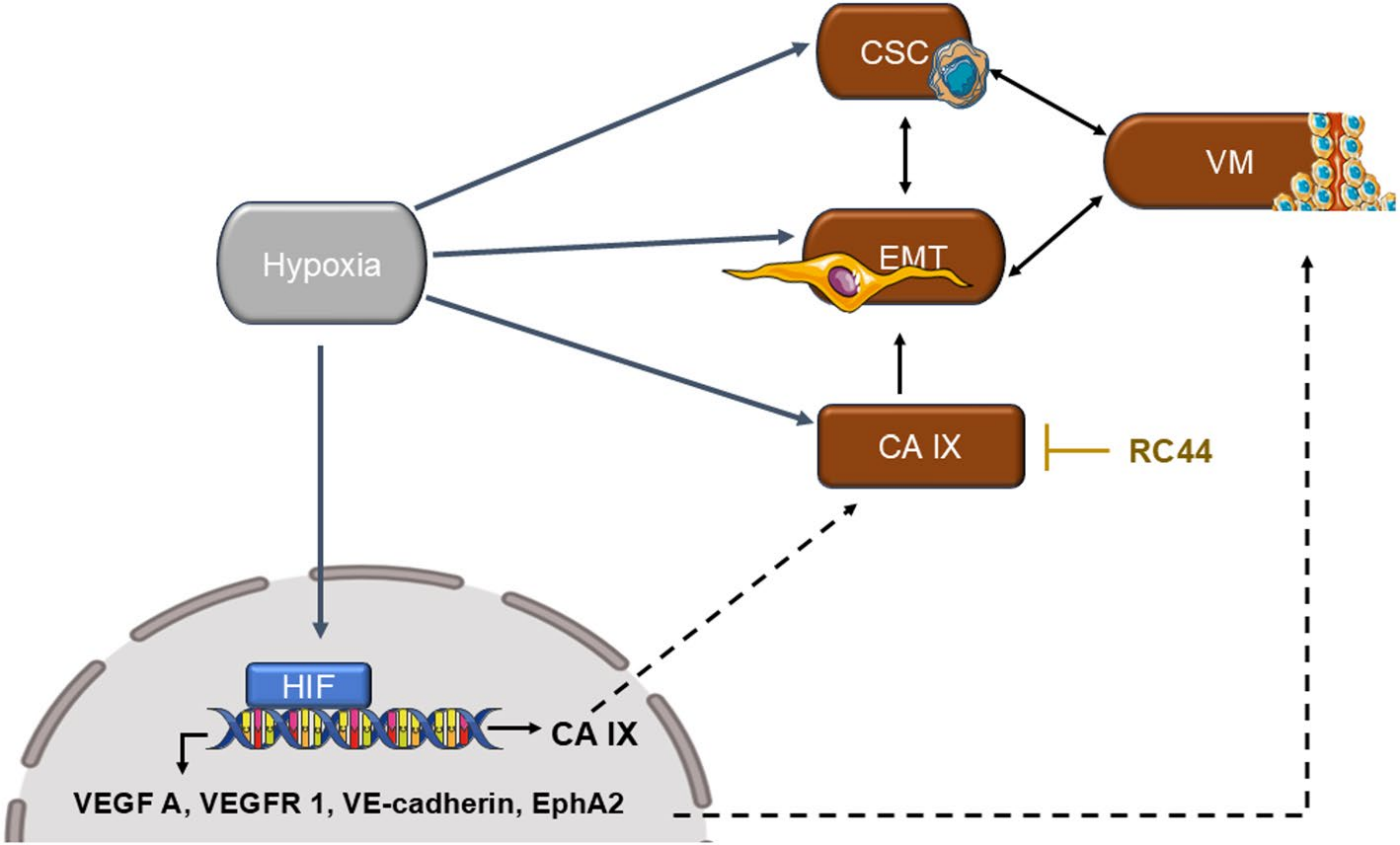
Schematic representation of hypoxia-induced vasculogenic mimicry. Hypoxia independently activates multiple parallel adaptive responses that converge to promote VM. It stabilizes hypoxia-inducible factors (HIFs), which drive the transcription of VM-associated genes (e.g., VE-cadherin, EphA2, VEGF-A), while concurrently inducing epithelial–mesenchymal transition (EMT), enhancing cancer stem cell plasticity, and upregulating carbonic anhydrase IX (CA IX) expression. These pathways interact as a network to facilitate VM formation under hypoxic stress. The figure was generated using drawing Tools. Abbreviations: CA IX, carbonic anhydrase IX; EphA2, ephrin type A receptor 2; EMT, epithelial-mesenchymal transition; HIF, hypoxia-inducible factor; VE-cadherin, vascular endothelial-cadherin; VEGF-A, vascular endothelial growth factor A

Hypoxia also drives EMT, enabling tumor cells to lose epithelial traits (E-cadherin) and acquire mesenchymal markers (vimentin, N-cadherin) [52, 53]. This transition is regulated by HIF-1α-mediated factors, including SNAIL, SLUG, TWIST 1, and ZEB, which collectively promote VM formation in breast cancer [52, 54]. Additionally, HIF-1α induces the expression of carbonic anhydrase (CA) IX, a pH-regulating enzyme, to drive EMT, CSCs, and VM formation in TNBC [52]. Together, these findings suggest that hypoxia integrates CSC and EMT pathways to drive VM (Fig. 2). Targeting HIF-1α or its downstream effectors, such as CA IX, may therefore suppress VM formation in hypoxic tumors.

Moreover, HIF-1α stabilization enhances VEGF-A expression, and the binding of VEGF-A/VEGFR-1 promotes VM formation in aggressive and highly invasive breast cancer cells [55]. While VEGFR-1 is most frequently linked to VM, other receptors such as VEGFR-2 and VEGFR-3 have also been implicated in VM across different breast cancer cell lines [34].

### Vasculogenic mimicry execution pathways

Vasculogenic mimicry channel formation depends on the coordinated activity of VE-cadherin, ephrin type A receptor 2 (EphA2), and downstream signaling cascades. VE-cadherin, a cell–cell adhesion molecule, co-localizes and interacts with EphA2, in the intercellular junctions between cells that form VM channels, leading to its phosphorylation [48]. Phosphorylated EphA2 then promotes the translocation of focal adhesion kinase (FAK) to new adhesive sites, as well as the subsequent activation of phosphoinositide 3 kinase (PI3K) signaling [17, 56, 57]. Activated PI3K enhances cell survival and proliferation and upregulates MMP activity, particularly MMP14, which in turn activates MMP2 and MMP9 [57]. These proteases remodel the extracellular matrix by cleaving the laminin-5γ2 chain, thereby promoting VM formation [39, 58].

**Fig. 3:**
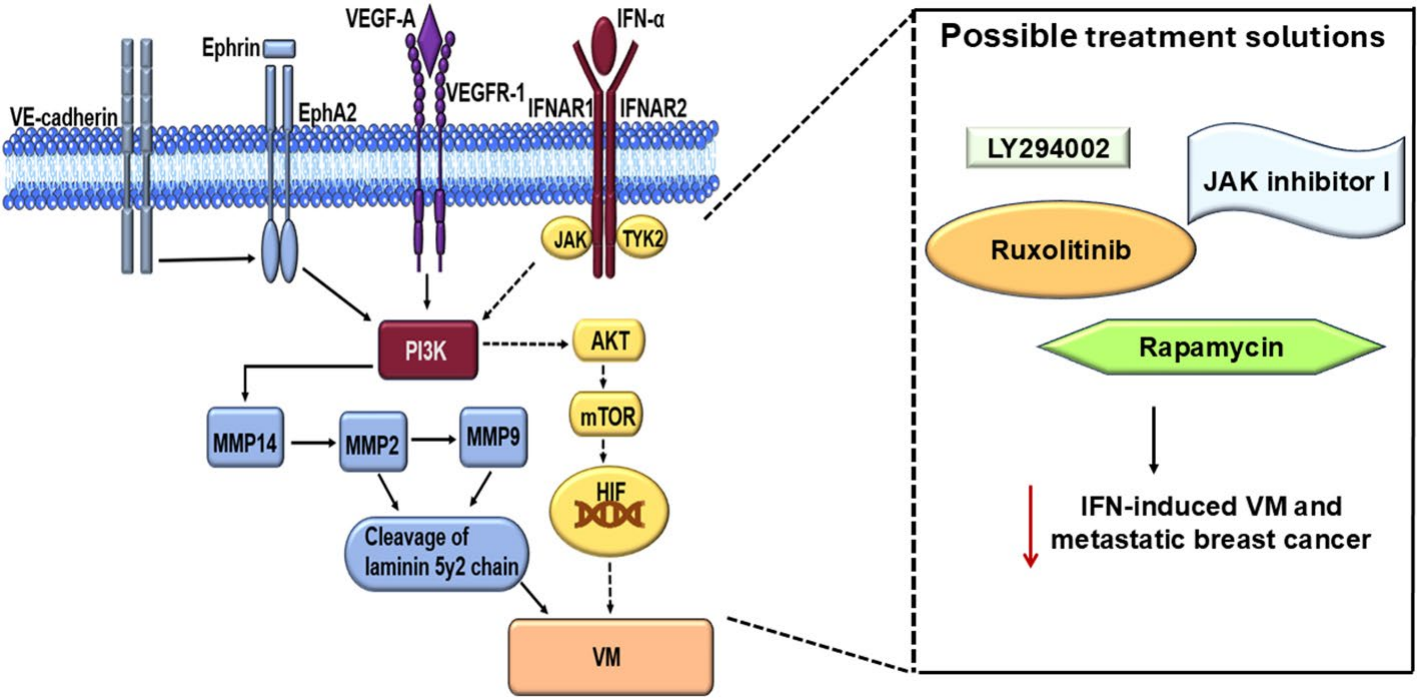
Major pathways contributing to vasculogenic mimicry in breast cancer. Ligand-receptor interactions, including VEGF-A/VEGFR-1, Ephrin/EphA2, and IFN-α/IFNAR 1/2, as well as VE-cadherin co-localization, activate PI3K signaling. This activation promotes upregulation of MMP2, MMP9, and MMP14, leading to cleavage of the laminin 5γ2 chain and VM formation. In parallel, IFN-α can activate JAK and TYK2, triggering the downstream AKT/mTOR/HIF-1α axis that may facilitate VM formation. Potential therapeutic inhibitors, LY294002 (PI3K), JAK inhibitor I, Ruxolitinib (JAK1/2), and Rapamycin (mTOR), are highlighted as possible strategies to suppress IFN-induced VM and breast cancer metastasis. Dashed arrows represent mechanistic links inferred from studies in other tumor types and not yet experimentally confirmed in breast cancer. Generated using drawing tools. Abbreviations: VEGF-A, vascular endothelial growth factor-A; VEGFR-1, vascular endothelial growth factor receptor-1; IFN-α, interferon-α; IFNAR 1/2, interferon alpha receptor 1 and 2; VE, vascular endothelial; PI3K, phosphoinositide 3-kinase; MMP, matrix metalloproteinases; JAK, Janus kinase; TYK2, tyrosine kinase 2; AKT, protein kinase B; mTOR, mammalian target of rapamycin; HIF, hypoxia-inducible factor

Inhibition studies further confirm the importance of this pathway. In a study conducted by Marouf et al., a combination treatment that inhibits angiogenesis, consisting of apatinib (a tyrosine kinase inhibitor) and melatonin (1µM/ 100mM), failed to suppress the expression of MMP14 in MDA-MB-231 cells. However, the inhibition of MMP2 and MMP9 reduced VM in the same cells [59]. Similarly, Knockdown of TRPS1, a GATA transcription factor, reduced the ability of breast cancer cells to form VM by downregulating MMP2 and MMP9 [60]. Metalloproteinase 9 also amplifies VEGF-A expression, which plays a role in both VM and angiogenesis [61]. Both of these processes are necessary for VM-mediated breast cancer metastasis, as VM channels form junctions with microvessels that are formed through angiogenesis. These findings underscore the PI3K/MMP axis as a key execution pathway and potential therapeutic target in VM (Fig. 3).

### Metabolic regulation of vasculogenic mimicry

Vasculogenic mimicry is also linked to tumor metabolic reprogramming. In p53-mutated TNBC, enhanced glucose metabolism supports the formation of tumor-lined pseudovessels (Fig. 1) [62]. Pyruvate kinase M2 (PKM2), a rate-limiting glycolytic enzyme, promotes VM by increasing glucose flux and elevating VE-cadherin expression, a key VM marker. Under normal conditions, phosphorylated checkpoint kinase 2 (Chk2) at T68 interacts with and phosphorylates PKM2 at Ser100, facilitating its nuclear export and reducing VM formation [62].

However, in p53-deficient tumors, overexpression of microRNA-191 (miR-191) suppresses Chk2 expression [63, 64], weakening its inhibitory control over PKM2. The resulting hyperactivation of PKM2 enhances glycolysis and VM formation. This Chk2–PKM2–miR-191 regulatory loop, therefore, links DNA damage response, metabolic adaptation, and VM. Restoring Chk2 function or inhibiting PKM2 may offer novel therapeutic strategies for suppressing VM in p53-mutated TNBC.

Together, these regulatory mechanisms underscore the multifactorial nature of VM in breast cancer. Hypoxia initiates a cascade that induces EMT and stem-like properties, while extracellular matrix remodeling and metabolic reprogramming provide the structural and energetic support for VM channel formation. These interconnected pathways suggest that VM is not a standalone process but a manifestation of tumor cell adaptability under stress. Given this complex regulation, microRNAs have emerged as key post-transcriptional regulators that can modulate VM-associated pathways, providing an additional layer of control over tumor adaptability.

## MicroRNA regulation of vasculogenic mimicry in breast cancer

MicroRNAs are small non-coding RNA molecules that regulate gene expression at both transcription and translation levels [65, 66]. These non-coding RNA molecules target messenger RNAs (mRNAs) at the post-transcription level by partially complementing with the 3’ untranslated region (UTR) of the mRNA, resulting in mRNA degradation and translation repression. In breast cancer, miRNAs are key modulators of tumor invasion, metastasis, and therapeutic resistance [67, 68]. Although only a few studies have investigated their specific involvement in VM, existing evidence demonstrates that miRNAs function as either positive (pro-VM) or negative (anti-VM) regulators, depending on the genes and pathways they target.

### Pro-vasculogenic mimicry microRNAs in breast cancer

Triple-negative breast cancer cells expressing high levels of miR-93 show enhanced proliferation and metastasis [69]. MicroRNA-93, an oncogenic member of the miR-106b-25 cluster, promotes EMT by increasing N-cadherin and vimentin expression while reducing E-cadherin and occluding levels, molecular changes that facilitate the phenotypic switch associated with VM formation [70, 71]. Thus, miR-93 may drive VM by enabling EMT-mediated plasticity in TNBC cells.

Similarly, miR-526b and miR-655 promote EMT, migration, and invasion in breast cancer [72, 73]. MCF-7 cells overexpressing these miRNAs form vessel-like tubular structures, confirming their role in VM [72]. Hypoxic exposure further enhances VM potential in miR-526b- and miR-655-overexpressing cells, highlighting the link between hypoxia-driven signaling and oncogenic miRNA expression.

Moreover, dysregulated expression of oncogenic miRNAs correlates with poor prognosis and aggressive tumor phenotypes in breast cancer [74]. These pro-VM miRNAs could therefore serve as potential therapeutic targets, particularly in aggressive subtypes where they sustain tumor adaptability and resistance. Conversely, several tumor-suppressive miRNAs act as inhibitors of VM formation, attenuating oncogenic signaling and remodeling within the tumor microenvironment.

### Anti-vasculogenic mimicry microRNAs in breast cancer

Tumor-suppressor miRNAs inhibit VM by targeting genes and signaling pathways that promote endothelial-like transformation, invasion, and therapeutic resistance. These anti-VM miRNAs function through diverse mechanisms, including suppression of pro-inflammatory, EMT-inducing, hypoxia-responsive, and metabolic regulators.

#### Targeting IL-6/STAT3 signaling

The IL-6/signal transducer and activator of transcription 3 (STAT3) pathway promotes VM formation by upregulating key regulators such as VE-cadherin and MMP2, while also contributing to chemotherapy resistance and tumor recurrence [75, 76]. Chemotherapeutic agents, such as cisplatin, induce IL-6 expression in endothelial cells, which in turn enhances VM through tumor–endothelial cell crosstalk [77, 78]. Upregulation of miR-125a and let-7e, which target IL-6, the IL-6 receptor, and STAT3, exerts anti-VM effects and sensitizes tumor cells to chemotherapy [78, 79]. Therefore, combining tumor-suppressive miRNAs with chemotherapeutic agents may counteract VM-associated chemoresistance.

#### Targeting AXL signaling

Under hypoxic conditions, the AXL receptor tyrosine kinase, activated by its ligand growth-arrest specific gene 6, drives EMT, metastasis, drug resistance, and VM [80–82]. Overexpression of AXL in MDA-MB-231 and MCF-7 cells promotes VM formation through EMT regulation. The tumor-suppressor miR-34a directly targets AXL, reversing EMT and reducing VM, invasion, and metastasis [82]. This highlights miR-34a as a critical anti-VM regulator in hypoxia-adapted breast tumors.

#### Targeting ZEB1 under nutrient stress

Tumor cells exposed to nutrient-deprived environments exhibit phenotypic switching toward mesenchymal states that favor VM. However, miR-200c, miR-96, and miR-182, which target the EMT regulator ZEB1, effectively suppress VM formation and downregulate associated promoters such as fibronectin 1, serine protease inhibitor family E member 2, low-density lipoprotein receptor-related protein 1, and integrin β1 [83]. These findings underscore the role of metabolic stress in VM induction and suggest that restoring ZEB1-targeting miRNAs could impair tumor adaptability under stress conditions.

#### Targeting HIF-1α/CREB5 signaling

The tumor suppressor miR-204 inhibits VM in TNBC by targeting HIF-1α and downstream signaling components, including PI3K/AKT, RAF1/MAPK, VEGF-A, and FAK/SRC pathways [35]. Additionally, research shows that miR-204 targets CREB5, a hypoxia-activated transcription factor associated with VM formation, as well as other key oncogenic effectors such as PI3K-α and c-SRC [35, 84]. Through this multitarget suppression, miR-204 effectively blocks VM and highlights the therapeutic advantage of miRNAs with broad regulatory capacity.

#### Other validated anti-vasculogenic mimicry MiRNAs

Beyond these major pathways, several other miRNAs directly target key VM drivers. MiR-299-5p inhibits VM by targeting osteopontin, a phosphoprotein that promotes spheroid-derived tumor cell transformation into vascular-like structures [85, 86]. MicroRNA-193b suppresses dimethylaminohydrolase (DDHA1), an enzyme involved in the metabolism of an endogenous inhibitor of nitric oxide synthase, asymmetric dimethylarginine and promotes VM in aggressive TNBC [87, 88]. Similarly, miR-340-5p targets Seven *in absentia* homolog 2 (SIAH2) in hypoxia-treated breast cancer stem-like side-population cells, suppressing EMT and VM [89].

Overall, these anti-VM miRNAs interfere with multiple oncogenic pathways, including IL-6/STAT3, AXL, ZEB1, and HIF-1α, underscoring the multifaceted regulatory landscape of miRNA-mediated VM control in breast cancer. Their multi-targeting potential and ability to restore tumor-suppressive signaling could make them promising candidates for combination therapies aimed at inhibiting VM and overcoming treatment resistance.

## Detection and quantification of vasculogenic mimicry in breast cancer

Accurate detection and quantification of VM are essential for understanding its prognostic significance and therapeutic implications in breast cancer. However, the absence of standardized diagnostic criteria has led to inconsistencies across studies. Detection typically involves a combination of histochemical, immunohistochemical, and molecular approaches, each offering unique advantages and limitations.

### Histological detection of vasculogenic mimicry

Vasculogenic mimicry is traditionally identified using PAS staining in combination with the absence of endothelial markers (CD31/CD34), a dual histochemical–immunohistochemical method first established by Maniotis et al. as the gold standard for distinguishing VM from classical angiogenesis [16, 90, 91]. Preclinical studies have since applied PAS/CD31 double-staining to various tumor tissues, including invasive ductal carcinoma specimens, confirming the presence of VM across multiple cancer subtypes [39, 41].

However, relying solely on PAS/CD31 staining may be misleading. PAS positivity is not entirely specific for VM, as glycogen- and glycoprotein-rich deposits in necrotic or fibrotic tumor regions can also stain with PAS, potentially confounding VM assessment [92, 93]. Therefore, PAS/CD31 (or CD34) dual staining should be interpreted cautiously and, where possible, complemented by additional VM-associated markers such as VE-cadherin, EphA2, and laminin 5γ2 to verify the tumor origin of VM channels.

While histological detection remains the cornerstone for VM identification, it primarily offers morphological evidence and lacks information about functional aspects such as blood perfusion and flow dynamics, underscoring the need for complementary functional imaging methods [92, 94].

### Functional imaging techniques for vasculogenic mimicry detection and characterization

To overcome the limitations of conventional histology, advanced imaging modalities have been developed to visualize VM *in vivo*. Standard techniques such as Doppler ultrasound and magnetic resonance imaging (MRI) can assess perfusion, permeability, and flow within tumor-associated vessels, helping identify VM-rich regions [91, 95]. However, these methods cannot specifically distinguish tumor cell–lined channels from endothelial vessels.

Contrast-enhanced ultrasound (CEUS), an advanced functional imaging approach, uses microbubble contrast agents to provide dynamic, real-time assessment of microcirculation, offering additional insight into VM activity beyond that provided by structural imaging. Nevertheless, CEUS reflects overall perfusion rather than VM-specific channels, and aligning imaging planes precisely with histological slices can be challenging, limiting direct correlation between functional and structural data [96].

Emerging modalities such as optoacoustic (photoacoustic) tomography combine optical and ultrasound imaging to provide high-resolution, non-invasive assessment of VM. In breast cancer xenograft models, optoacoustic imaging has successfully differentiated VM from angiogenic vessels by detecting variations in oxygenation and vessel maturity, highlighting its promise for functional characterization of vascular phenotypes in clinical settings [97].

Overall, the accuracy of imaging-based VM detection depends on spatial resolution and contrast specificity. Because histology provides structural information while functional imaging reveals dynamic perfusion, combining these approaches with molecular markers remains essential for reliable and comprehensive identification of VM.

### *In vitro *assays for vasculogenic mimicry detection and quantification

Functional and morphological studies of VM can also be performed *in vitro* using 3D culture assays coupled with microscopy [98]. These assays allow researchers to observe endothelial-like channel formation, assess structural properties, and investigate molecular regulators under controlled conditions.

For over a decade, VM structures were quantified manually because dedicated analysis tools were lacking. Existing angiogenesis software is often incompatible with the unique morphology of VM networks, resulting in inaccurate measurements. Manual quantification is also time-consuming and prone to bias [92, 99].

To address these limitations, Moore et al. developed a VaMiAnalyzer, a stand-alone software that enables automated measurement of VM structural features from phase-contrast microscopy images, providing more accurate and efficient quantification of vessel-like networks [99].

### Bioinformatic identification of vasculogenic mimicry related genes and signatures

Beyond histological detection, bioinformatic analyses have become essential for elucidating the molecular mechanisms and clinical implications of VM in breast cancer. These techniques integrate gene expression profiling, single-cell sequencing, pathway enrichment, and spatial transcriptomic analyses to identify VM-related genes and molecular subtypes, thereby supporting prognostic modeling and therapeutic prediction [100, 101].

Zheng et al. utilized transcriptomic, single-cell sequencing, and clinical data of breast cancer patients from The Cancer Genome Atlas (TCGA) and Gene Expression Omnibus (GEO) databases to perform single-cell annotation and spatial expression analysis to identify VM-related genes [100]. The study constructed VM-related biomarkers and a prognostic model, explored their association with immune infiltration, and investigated their role in tumor invasion and response to immunotherapy, highlighting their potential in patient stratification and personalized treatment planning. Similarly, Li et al. used machine learning algorithms on large-scale bulk and single-cell datasets with experimental validation via immunohistochemistry, resulting in a novel VM gene signature that predicts breast cancer prognosis and guides potential therapeutic strategies [102].

In parallel, bioinformatic analyses can delineate key molecular determinants of VM. Notable candidates include VE-cadherin (CDH5), EPHA2, TWIST1, MMP2, and Laminin 5γ2 (LAMC2), all of which are implicated in cell plasticity, migration, and extracellular matrix remodeling [17, 101]. Pathway enrichment analyses, such as Gene Ontology (GO), can further contextualize these genes within biological pathways relevant to VM, including EMT, PI3K/AKT, and hypoxia signaling [101, 103].

Collectively, these bioinformatic advances provide a foundation for developing VM-based prognostic models and personalized therapeutic strategies in breast cancer.

## Therapeutic strategies that inhibit vasculogenic mimicry in breast cancer

While VM is increasingly recognized as a contributor to tumor progression and therapeutic resistance in breast cancer, there are currently no approved clinical agents that directly target this process. Nevertheless, several experimental and pathway-targeted molecules have shown promise in disrupting VM formation by modulating its underlying molecular and cellular mechanisms.

### Emerging therapeutic agents and pathway inhibitors

The small-molecule compound CVM-1118 (foslinanib) is a novel anti-VM agent currently undergoing Phase IIa clinical evaluation (NCT05257590, NCT03600233) for non-breast malignancies [104, 105]. Preclinical studies demonstrate that CVM-1118 inhibits VM by targeting the mitochondrial chaperone TRAP1, leading to destabilization of HIF-1α and suppression of VM-associated gene expression (such as VEGF-A, VEGFR-1, and EphA) in 3D and xenograft models [106]. Although these results derive from non-breast tumor systems, the shared hypoxia-HIF signaling context suggests potential translational relevance for breast cancer, which remains to be experimentally validated.

Independently, interferon-α (IFN-α) signaling has been shown to activate the JAK1/PI3K/AKT/mTOR cascade, which enhances HIF-1α-mediated EMT and promotes VM formation. Pharmacologic inhibition of this axis using JAK inhibitor I, LY294002, or Rapamycin significantly suppresses IFN-induced VM formation in renal carcinoma cells [107]. In the same study, IFN-α also increased HIF-1α expression in MCF-7 and MDA-MB-231 breast cancer cells, suggesting that components of this signaling network are present and responsive in breast cancer. Although the full pathway has not yet been functionally validated in this context, such IFN-α–HIF-1α induction may contribute to tumor cell plasticity potentially relevant to VM **(**Fig. 3**)**.

### Natural compounds and nanocarrier approaches

In addition to synthetic small molecules, various natural compounds have been explored for their ability to inhibit VM in breast cancer. Preclinical studies using in vitro breast cancer cell models and in vivo xenograft models have demonstrated that natural compounds such as Xian-ling-lian-xia-fang and Sinomenine may inhibit VM by downregulating VM-associated pathways, including VEGF-A/MMP2 and the miR-340-5p/SIAH2 axis, respectively [89, 108]. However, the translation of these natural compounds to clinical drugs is often accompanied by bioavailability and toxicity challenges [109, 110]. Due to their complex and diverse structures, these natural compounds are often poorly absorbed and rapidly metabolized in the human body, which affects their therapeutic effect. Drug delivery systems, such as nanoparticles and liposomes, may be useful in improving the bioavailability of the natural products [109]. In a study conducted by Ju et al., Hyaluronic acid-modified daunorubicin plus honokiol cationic liposomes increased intracellular accumulation and destroyed VM by down-regulating VM protein indicators (FAK, EphA2, MMP-2, and MMP-9) in MCF-7 cells, MDA-MB-435S cells, and xenografts of MDA-MB-435S cells [111].

Beyond single-agent natural compounds, nanocarrier-based combination therapies have demonstrated promise. Co-delivery of vincristine, a vinca alkaloid with antiangiogenic activity, and dasatinib, a multi-tyrosine kinase inhibitor targeting c-KIT, Platelet-Derived Growth Factor Receptor (PDGFR)-α/β, ephrin receptor kinases, ABL, and SRC, via liposomes significantly inhibited VM in MDA-MB-231 cells [46, 57, 112]. Liposomal encapsulation enhances the therapeutic index by accommodating both hydrophilic and hydrophobic drugs, thereby improving tumor targeting and reducing systemic toxicity [57]. These findings underscore the potential of nanocarrier-mediated combination strategies to overcome resistance and target VM, providing a preclinical rationale for incorporating such approaches into precision oncology strategies for breast cancer.

### Precision oncology strategies guided by bioinformatics

Inter-patient variability in response to therapy, even among similar molecular subtypes, highlights the need for precision-based interventions. These differences often arise from individual genetic variations that influence tumor behavior and mutation profiles [113, 114]. In a study by Cannell et al., bioinformatic tools were used to show that FOXC2-driven gene expression contributes to resistance to bevacizumab, an anti-angiogenic drug, via VM mechanisms in breast cancer patients [19]. This finding highlights how bioinformatics can support the detection of differential therapeutic responses and advance the implementation of precision oncology.

Building on the VM gene signatures described in Sect. 5.4, precision oncology leverages these molecular markers to stratify patients and guide treatment decisions. By identifying individuals with VM-associated gene signatures, this approach can: (i) enhance the effectiveness of trials by focusing on those most likely to benefit from VM-targeted treatments; (ii) pinpoint patients who may be more resistant to standard anti-angiogenic therapies, supporting consideration of alternative treatment options; and (iii) support adaptive trial designs, where molecularly defined groups can lead to modifications of treatment arms as the study progresses [19, 115, 116].

Despite several challenges that hinder the routine application of VM gene signatures in clinical practice, including variability in sample preparation, inconsistencies in analysis pipelines that affect reproducibility, and limited clinical validation due to the scarcity of available VM gene signatures, these obstacles are being addressed through emerging technologies. Techniques such as spatial transcriptomics provide valuable spatial context to VM gene expression, while advances in artificial intelligence (AI) facilitate improved pattern recognition and integration of diverse data types [102, 117]. Collaborative initiatives aimed at standardizing bioinformatic pipelines and compiling large, well-annotated clinical datasets are essential for transitioning VM gene signatures.

## Translational challenges and preclinical considerations for vasculogenic mimicry in breast cancer

### Limited clinical translation and diagnostic standardization

**Fig. 4:**
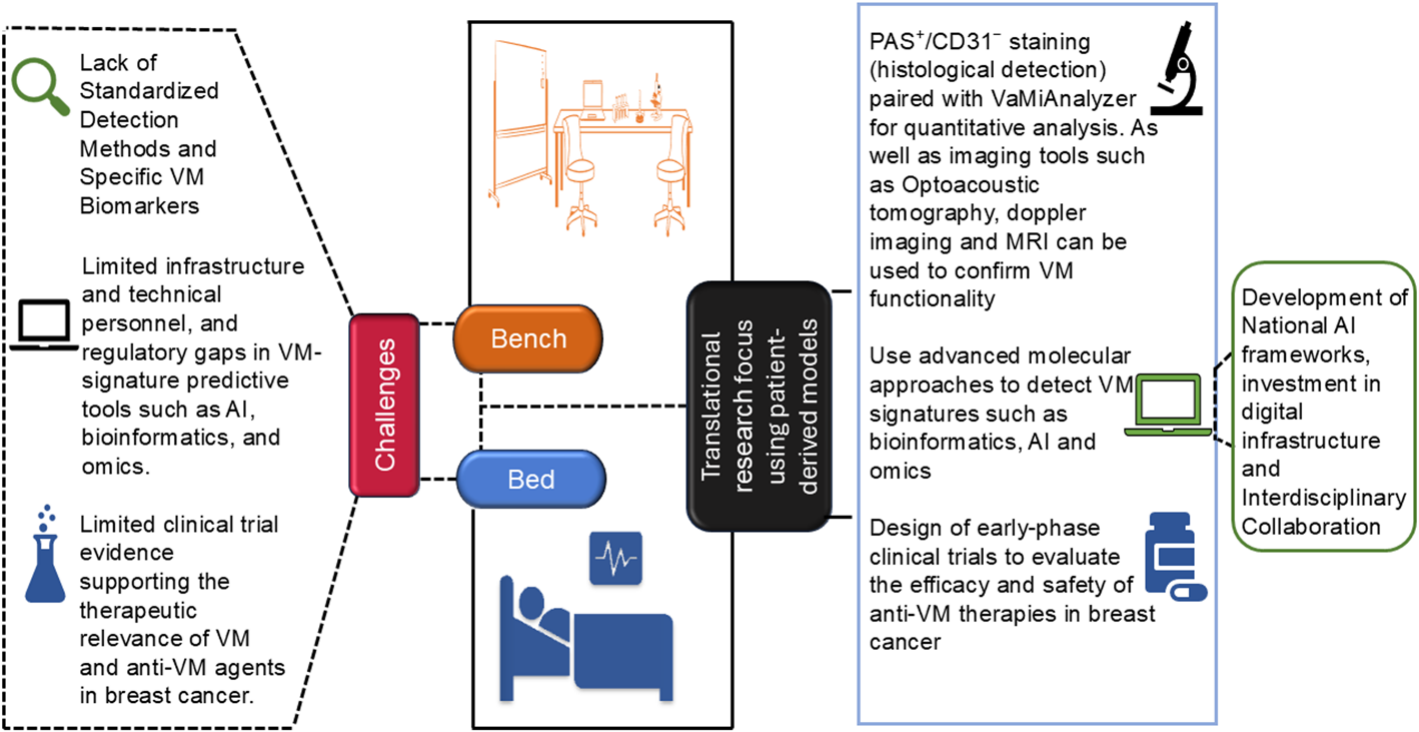
Bridging Vasculogenic Mimicry Therapy from Bench to Bedside. Schematic illustration of the translational journey of VM-targeted therapies. On the left, key barriers are presented, including the lack of standardized detection methods, limited analytic infrastructure for high-throughput technologies, and the scarcity of clinical trials investigating the efficacy of anti-VM therapies. The center depicts essential components of the translational bridge, including patient-derived models, robust histological tools such as PAS⁺/CD31⁻ staining integrated with platforms like VaMiAnalyzer, along with advanced imaging, molecular profiling, and biomarker-guided early-phase trials that bring laboratory findings closer to clinical application. On the right, strategic enablers aimed at strengthening predictive tools for VM, including the development of national AI policies, improved infrastructure, and fostering interdisciplinary collaboration to support effective clinical translation. Generated using drawing tools

Vasculogenic mimicry has been associated with poor prognosis and therapeutic resistance in breast cancer [18, 118, 119]. Despite extensive preclinical research linking VM to tumor aggressiveness and relapse, its clinical translation remains a challenge. There is a lack of clinical trials designed to evaluate anti-VM effects in breast cancer, restricting the understanding of how preclinical research drugs may perform in patients. A major barrier is the lack of standardized clinical methods for detecting and quantifying VM, which complicates the selection of eligible participants for potential trials (Fig. 4**)**. Differentiating VM from angiogenesis remains a major diagnostic obstacle, underscoring the need for unified imaging, molecular, and histopathological criteria for clinical assessment.

### Preclinical models

Preclinical models have provided invaluable insights into VM biology, yet they only partially recapitulate the complexity of human tumors. Traditional 2D cultures lack the 3D architecture and microenvironmental diversity necessary to sustain physiologically relevant VM structures. Although 3D cultures and xenografts improve physiological relevance, they still have limitations, particularly in reproducing human stromal–immune–tumor interactions. Furthermore, many preclinical studies rely on cell-line-derived xenografts that may not reflect the genetic and phenotypic diversity of patient tumors [45, 108, 120]. The integration of patient-derived organoids, tumor-on-a-chip systems, and co-culture platforms could bridge this gap, offering more predictive and reproducible models for VM-targeted drug testing [121, 122].

Bridging the preclinical–clinical gap in VM research will likely necessitate a dual strategy: (i) developing standardized diagnostic criteria and imaging algorithms for detecting VM in patient tissues, and (ii) employing physiologically relevant models to evaluate emerging anti-VM agents. Accordingly, fostering cross-disciplinary collaborations among pathologists, computational biologists, and translational oncologists will be critical to expediting clinical validation.

## Conclusion

Vasculogenic mimicry is an adaptive mechanism in which breast tumors maintain perfusion without endothelial cell involvement. This process enables tumors to endure hypoxic environments, contributes to resistance against therapies, and facilitates both metastasis and recurrence. Accumulating evidence over the past two decades supports VM as a relevant feature of aggressive disease, particularly in triple-negative and HER2-enriched subtypes. Despite this, clinical translation remains limited due to heterogeneous presentation, overlap with classical angiogenesis markers, and a lack of standardized detection methods. Integrative diagnostic frameworks that combine morphological, molecular, and functional assessments are therefore critical for accurately identifying VM and assessing its clinical relevance.

Emerging technologies, including AI-assisted histopathology, multi-omics integration, spatial transcriptomics, and nanocarrier-based drug delivery, offer promising avenues to overcome these challenges. Multi-targeted therapeutic strategies that disrupt VM, angiogenesis, and tumor adaptive networks simultaneously have the potential to enhance treatment efficacy and reduce resistance. Incorporating VM assessment as an exploratory biomarker in clinical trials may be valuable for establishing its prognostic and predictive significance.

Ultimately, progress in VM-directed therapy will likely rely on coordinated interdisciplinary collaboration, methodological standardization, and expanded global research efforts aimed at bridging the gap between experimental findings and clinical application.

## Data Availability

No datasets were generated or analysed during the current study.

## Abbreviations

AI: Artificial intelligence
CA: Carbonic anhydrase
CEUS: Contrast-enhanced ultrasound
Chk2: Checkpoint kinase 2
CSCs: Cancer stem-like cells
DDHA1: Dimethylarginine dimethylaminohydrolase 1
EMT: Epithelial-mesenchymal transition
EphA: Erythropoietin-producing human hepatocellular receptor A2
ER: Estrogen receptor
FAK: Focal adhesion kinase
GEO: Gene Expression Omnibus
GO: Gene ontology
HER2: Human epidermal receptor 2
HIF-1α: Hypoxia inducible factor-1α
IARC: International Agency for Research on Cancer
IFN: Interferon
IFNAR: 1/2 interferon alpha receptor 1 and 2
IL: Interleukin
JAK: Janus kinase
MiR: MicroRNA
mRNAs: Messenger RNAs
MMP: Matrix metalloproteinase
mTOR: Mammalian of rapamycin
PDGFR: Platelet-derived growth factor receptor
PI3K: Phosphoinositide 3 kinase
PKM2: Pyruvate kinase M2
PR: Progesterone receptor
SIAH2: Seven in absentia Homolog 2
STAT3: Signal transducer and activator of transcription 3
TCGA: The Cancer Genome Atlas
TGF-β: Transforming growth factor-β
TNBC: Triple negative breast cancer
TNF-α: Tumor necrosis factor
UTR: Untranslated region
VE: Vascular endothelial
VEGF: Vascular endothelial growth factor
VEGFR: Vascular endothelial growth factor receptor
VM: Vasculogenic mimicry
